# Carriage of Methicillin-Resistant *Staphylococcus aureus* by Wild Urban Norway Rats (*Rattus norvegicus*)

**DOI:** 10.1371/journal.pone.0087983

**Published:** 2014-02-03

**Authors:** Chelsea G. Himsworth, Ruth R. Miller, Vincent Montoya, Linda Hoang, Marc G. Romney, Ghada N. Al-Rawahi, Thomas Kerr, Claire M. Jardine, David M. Patrick, Patrick Tang, J. Scott Weese

**Affiliations:** 1 School of Population and Public Health, University of British Columbia, Vancouver, British Columbia, Canada; 2 Animal Health Centre, British Columbia Ministry of Agriculture, Abbotsford, British Columbia, Canada; 3 Department of Pathology and Laboratory Medicine, University of British Columbia, Vancouver, British Columbia, Canada; 4 British Columbia Centre for Disease Control, Vancouver, British Columbia, Canada; 5 Department of Medicine, University of British Columbia, Vancouver, British Columbia, Canada; 6 St. Paul's Hospital, Vancouver, British Columbia, Canada; 7 Children's and Women's Health Centre of British Columbia, Vancouver, British Columbia, Canada; 8 British Columbia Centre for Excellence in HIV/AIDS, St. Paul's Hospital, Vancouver, British Columbia, Canada; 9 Department of Pathobiology, University of Guelph, Guelph, Ontario, Canada; Rockefeller University, United States of America

## Abstract

Methicillin-resistant *Staphylococcus aureus* (MRSA) is an important cause of multi-drug-resistant infections in people, particularly indigent populations. MRSA can be transmitted between people and domestic animals, but the potential for transmission between people and commensal pests, particularly rodents, had not been investigated. The objective of this study was to identify the presence and characterize the ecology of MRSA in rats (*Rattus* spp.) from in an impoverished, inner-city neighborhood. Oropharyngeal swabs were collected from rats trapped in 33 city blocks and one location within the adjacent port. Bacterial culture was performed and MRSA isolates were characterized using a variety of methods, including whole-genome sequencing (WGS). The ecology of MRSA in rats was described using phylogenetic analysis, geospatial analysis, and generalized linear mixed models. MRSA was identified 22 of 637 (3.5%) rats tested, although prevalence varied from 0 – 50% among blocks. Isolates belonged to 4 clusters according to WGS, with the largest cluster (n = 10) containing isolates that were genetically indistinguishable from community-acquired USA300 MRSA strains isolated from people within the study area. MRSA strains demonstrated both geographic clustering and dispersion. The odds of an individual rat carrying MRSA increased with increased body fat (OR = 2.53, 95% CI = 1.33 – 4.82), and in the winter (OR = 5.29, 95% CI = 1.04 – 26.85) and spring (OR = 5.50, 95% CI = 1.10 – 27.58) compared to the fall. The results show that urban rats carried the same MRSA lineages occurring in local human and/or animal populations, supporting recent transmission from external sources. MRSA carriage was influenced by season, most likely as a result of temporal variation in rat behavior and rat-human interactions.

## Introduction

*Staphylococcus aureus* is a gram-positive bacterium that colonizes epithelial surfaces and causes infections in humans [1]. Methicillin-resistance is mediated by *mecA* and related genes, which are carried on mobile genetic elements and confer resistance to most beta-lactam antimicrobials, and frequently other antimicrobial classes [1]–[3].

Since its emergence, methicillin-resistance *S. aureus* (MRSA) has become a significant cause of hospital-associated infections worldwide [1]. The early 2000s saw the emergence of community-associated MRSA (CA-MRSA), which, in contrast to hospital-associated MRSA (HA-MRSA), spreads and causes disease in the general population, outside of the healthcare setting and often in people without typical risk factors [1], [3]. CA-MRSA is particularly prevalent in North America, where it is an important cause of skin and soft tissue infections [1], [3].

Although colonization with CA-MRSA is widespread, the incidence of disease is greater in homeless people and injection drug users (IDUs) compared to the general population [1], [4], [5]. This is likely the result of a combination of factors including compromised health, crowding in shelters, poor skin integrity, and injecting in unhygienic environments [4], [5]. Indeed, soft tissue infections, including those caused by MRSA, account for the majority of hospitalizations among IDUs in some settings [6].

Although transmission of MRSA is primarily person-to-person, there is evidence that MRSA can be spread between domestic animals and people [7], [8]. Recently, questions have emerged regarding whether pest species might also be a source of MRSA [9]. The potential for pest-to-human MRSA transmission is particularly concerning in impoverished, inner-city neighborhoods, where factors associated with poverty may promote pest infestations and pest-human contact, and increase susceptibility to MRSA infection [5], [10].

Norway rats (*Rattus norvegicus*) are among the most common urban pest species and are known to be the source of a variety of zoonotic pathogens [10]. However, very little is known about MRSA in rats. One study demonstrated carriage of livestock-associated (LA-) MRSA in rats living on pig farms in the Netherlands [11], while another identified antibiotic-resistant *S. aureus* in black rats trapped in downtown-Tokyo, although these isolates were not definitively identified or characterized as MRSA [12].

Interestingly, methicillin-resistant *Staphylococcus pseudintermedius* has been identified in rats in the impoverished, inner-city Downtown Eastside (DTES) neighborhood of Vancouver, Canada [13], and MRSA was isolated from bedbugs in the same area [9]. The DTES is also home to a significant population of IDUs with a high prevalence of MRSA carriage [14] and infection [4], suggesting that this area should be a priority for the study of potential urban pest reservoirs of MRSA.

The objective of this study was to characterize the epidemiology of MRSA in rats from an inner-city neighborhood using culture, *spa* typing, antimicrobial susceptibility testing, and whole-genome sequencing, as well as data on rat distribution and demographic characteristics.

## Methods

### Ethics Statement

This study was approved by the University of British Columbia's Animal Care Committee (A11-0087) and adhered to national guidelines set out by the Canadian Council on Animal Care (www.ccac.ca), including those pertaining to animal user training, euthanasia, protocol review, and wildlife (http://www.ccac.ca/en_/standards/guidelines). This study did not involve any endangered or protected species.

### Sample Collection

The study area was comprised of 33 city blocks within the DTES (N49°17′/W123°6′) and 1 location within an international shipping port that forms the northern border of the DTES. Within the city blocks, trapping took place on public property only and no specific permissions were required. The port site was a private property that wishes to remain anonymous. Permission to trap at this site was obtained from the property manager.

Each block (and the port site) was assigned to a randomly selected 3 week study period between September 2011 and May 2012. Within each block, approximately 20 Tomahawk Rigid Traps for rats (Tomahawk Live Trap, Hazlelhurst, WI, USA) were set out along the back alley that bisected the block. Traps were pre-baited for 1 week, prior to 2 weeks of active trapping. At the port, traps were placed in areas where port staff had observed rats. Trapped rats were anesthetized with isoflurane prior to pentobarbital euthanasia. Immediately after euthanasia, a sterile swab was used to sample the oropharynx and nares of each rat.

Morphometric data collected in the field included species, sex, weight, nose-to-rump length, and sexual maturity (females with an open vaginal orifice and males with scrotal testes). The date and location (block and trap) of each rat trapped was also recorded. Rats subsequently underwent a full necropsy at the Animal Health Centre (AHC), British Columbia Ministry of Agriculture, Abbotsford, British Columbia, at which time sex and sexual maturity were confirmed and each rat received a score based on the volume of internal fat [poor condition (score of 0)  =  minimal to no visible internal fat; moderate condition (score of 1)  =  moderate internal fat; good condition (score of 2)  =  abundant internal fat].

### MRSA culture, *spa* typing, PVL PCR, and antibiotic susceptibility

Swabs were placed in 2 mL of enrichment broth containing 10 g/L tryptone T, 75 g/L sodium chloride, 10 g/L mannitol, and 2.5 g/L yeast extract and incubated for 24 h at 35°C. Aliquots of 100 µL were streaked onto MRSA Chromogenic agar (BBL CHROMagar, Becton, Dickinson and Co., Sparks, MD, USA) and incubated at 35°C for 48 h. Tube coagulase-positive isolates were speciated by using a multiplex PCR [15]. Methicillin resistance was confirmed by demonstrating the presence of penicillin-binding protein 2a antigen using a latex-agglutination test (Oxoid Ltd., Basingstoke, UK). MRSA isolates were characterized by *spa* typing [16] using the Ridom SpaServer (http://SpaServer.ridom.de). The *luk*F-PV gene encoding the Panton-Valentine leukocidin toxin was detected by real-time PCR [17]. Antimicrobial drug susceptibility was performed by broth microdilution (Sensititre, Trek Diagnostics, Cleveland, OH, USA), and results were interpreted according to Clinical and Laboratory Standards Institute guidelines [18].

### MRSA whole-genome sequencing

Total DNA was extracted from the rat MRSA isolates using the Qiagen DNeasy kit (Qiagen, Toronto, ON, Canada) and indexed DNA fragment libraries were prepared using the Nextera XT kit (Illumina, San Diego, CA, USA) as follows. After RNase treatment, DNA was quantitated in the QuBit (Life Technologies, Burlington, ON, Canada) and diluted to 0.2 ng/µl. For the Nextera XT kit, 5 µl of each sample was used as input for tagmentation followed by a low cycle number PCR reaction for indexing and adapter incorporation. Eight picomoles of each library was then quantified using a KAPA library quantification kit and loaded onto an Illumina MiSeq to generate 250 bp paired-end reads. Each library produced a mean of ∼560,000 paired-end reads with 53-fold coverage of the MRSA genome.

To enable a comparison of genetic diversity, sequences from 33 human USA300 (ST8/*spa* t008) isolates were also included for analysis. These isolates were collected as part of previous studies and sequenced on the Illumina HiSeq at Canada's Michael Smith Genome Sciences Centre using methods described previously [19]. These included 15 isolates from the DTES [4], [14] and 18 isolates from elsewhere within Vancouver (unpublished). Due to the large number of reads produced by the HiSeq, a subsample of 10% of reads was randomly selected from all human MRSA samples for use in downstream analysis, resulting in 2,200,000 base paired-end reads (2×75 bp) with 89-fold coverage of the MRSA genome for the human MRSA isolates.

Sequence reads were assembled using a pipeline specifically developed for bacterial genomes [20], modified for *S. aureus* by mapping to the USA300-FPR3757 reference genome [21], and estimated to have a false positive rate of 2.5×10^−9^ per nucleotide, i.e. 0.0075 per genome [22]. A mean of 92% and 93% of reads were mapped, resulting in 92% and 99% coverage of the USA300 reference genome for rat and human samples respectively before quality filtering. After filtering, calls made for a mean of 90% and 94% of the USA300 reference for the rat and human samples, respectively. One rat sample was excluded since all positions failed the filter due to poor sequence quality.

### Bioinformatics

To determine phylogenetic relationships among isolates, maximum-likelihood trees were generated from the entire genome sequence using PhyML [23] with the HKY85 substitution model, assuming a homogeneous mutation rate, with 500 bootstrap replicates.

In order to estimate the relatedness of the rat and human MRSA isolates, the average pairwise diversity (Π) was determined for all samples within (Π_x_) and between (Π_xy_) groups by calculating the sum of the total number of variant positions among samples in or between each group under consideration and then dividing that figure by the total number of samples.

To investigate the presence of mobile genetic elements potentially acquired from other bacterial species, the accessory genome was searched for all non-*S. aureus* elements using Velvet [24] in conjunction with the VelvetOptimiser (http://bioinformatics.net.au/software.velvetoptimiser.shtml). Reads that did not map to the USA300-FPR3757 reference were assembled and all contigs >1 kb were matched to the NBCI nr database using BLASTn. Any contig for which all matches with a BLAST score >95% of the maximum BLAST score for that contig were not *S. aureus* was considered potential non-*S. aureus*.

For in silico detection of the PVL gene and determination of the *spa* type from the genome sequence data, the VelvetOptimiser was used to assemble all the reads, after which the PVL and *spa* typing laboratory primers were used as queries against the contigs. Contigs containing any of the primers were selected for further analysis. PVL was deemed present if both PVL primers matched with no mismatches to the same contig, or to two contigs with an overlapping region which could be joined. *spa* type was determined by extracting the region between the two *spa* primers from a contig (after manual joining of two contigs if necessary) and entering the resulting sequence into the Ridom SpaServer.

### Statistical analysis

For the statistical analysis, the primary outcome variable was MRSA status (positive vs. negative). Explanatory variables that were considered included season (September – November  =  fall; December – February  =  winter; March – May  =  spring; June – August  =  summer), weight (per 10 g), length (cm), sex, sexual maturity (immature vs. mature), and fat score (score of 0 – 3).

Bivariable relationships between the explanatory and outcome variables were assessed using simple logistic regression, and explanatory variables associated with MRSA-status at p-≤0.10 were considered for inclusion in a generalized linear mixed model controlling for clustering by block of origin. For multivariable models, individuals with missing data for one or more of the variables under study were excluded. The final model was selected using Aikake's Information Criterion (AIC) to balance model fit and parsimony. All statistical analyses were conducted using R (R Development Core Team, Vienna, Austria).

### Spatial analysis

The location of each trap, the number of rats caught in each trap, and the number of rats that were MRSA-positive (including WGS group) were mapped using ArcGIS 10.0 (ESRI, Redlands, CA). This information was imported into SaTScan (Boston, USA) for cluster analysis using a purely spatial Bernoulli model and scanning for areas with high rates of MRSA using a circular window with a maximum spatial cluster size of 50% of the population at risk. Separate analyses were conducted for each MRSA WGS group vs. rats negative for that group (i.e., MRSA-negative rats and rats carrying other strains of MRSA). Clusters identified by SaTScan were visualized in ArcGIS.

## Results

### MRSA culture and Statistical analysis

**Figure 1:**
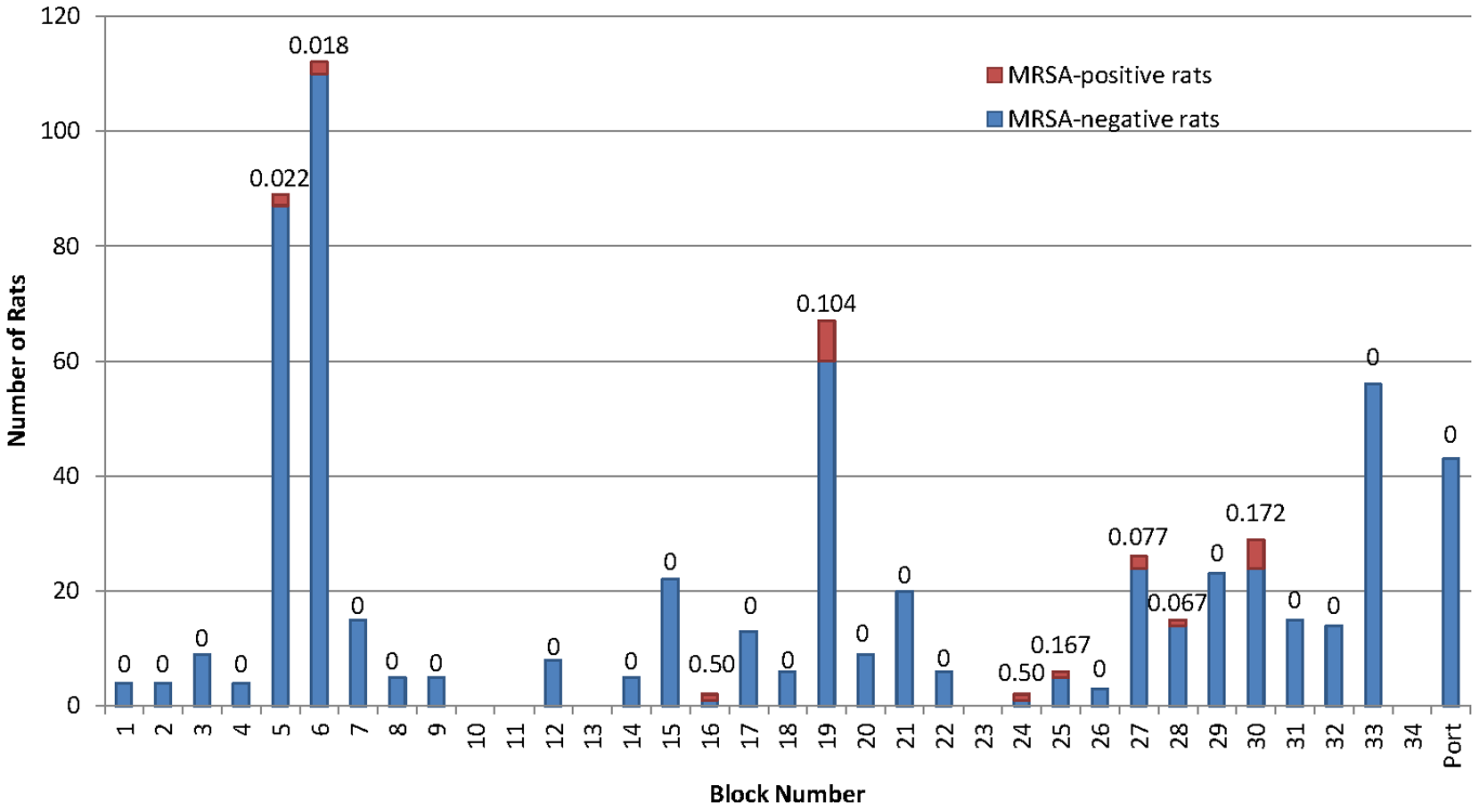
Prevalence of MRSA-positive and -negative rats in each city block.

A total of 637 rats were trapped and tested for MRSA carriage. Of these, MRSA was isolated from 22 rats (3.5%) (Table 1), although the prevalence of MRSA varied by block (Figure 1).

**Table 1:** Characteristics of 22 MRSA isolated obtained from wild Norway rats.

Rat number	Block	Trap	Date	Cluster	*spa* type	MLST	PVL	Ampicillin	Cefoxitin	Chloramphenicol	Ciprofloxacin	Clindamycin	Daptomycin	Erythromycin	Gentamicin	Levofloxacin	Linezolid	Moxifloxicin	Nitrofurantoin	Oxacillin +2% NaCl	Penicillin	Quinupristin/dalfopristin	Rifampin	Streptomycin	Tetracycline	Tigec ycline	Trimethoprim/sulphamethoxazole	Vancomycin
M11-82	05	018A	11/10/11	1	t008	8	Pos	>8^d^	>6	16	≤1	≤0.5	≤0.5	>4	≤2	>4	4	2	≤32	>4	>8	≤0.5	≤0.5	≤1000	≤2	0.25	≤0.5	1
M11-86	06	010	02/11/11	1	t008	8	Pos	>8	>6	16	>2	>2	≤0.5	>4	≤2	>4	4	2	≤32	>4	>8	1	1	≤1000	≤2	0.25	≤0.5	2
M12-83	16	006	10/02/12	1	t008	8	Pos	>8	>6	16	>2	≤0.5	≤0.5	>4	≤2	>4	4	2	≤32	>4	>8	≤0.5	1	≤1000	≤2	0.25	≤0.5	2
M12-84	19	002	10/02/12	1	t008	8	Pos	>8	>6	8	>2	≤0.5	≤0.5	>4	≤2	>4	4	2	≤32	>4	>8	≤0.5	≤0.5	≤1000	>16	0.25	≤0.5	1
M12-118	19	004	16/02/12	1	t008	8	Pos	>8	>6	8	>2	1	1	0.5	≤2	>4	2	2	≤32	>4	>8	≤0.5	2	≤1000	≤2	0.25	≤0.5	1
M12-119	19	012	16/02/12	1	t008	8	Neg	>8	>6	16	>2	1	≤0.5	>4	≤2	4	≤1	1	≤32	>4	>8	≤0.5	≤0.5	≤1000	≤2	0.25	≤0.5	1
M12-121	19	003	17/02/12	1	t818	8	Pos	>8	>6	8	>2	2	≤0.5	0.5	≤2	>4	2	2	≤32	>4	>8	≤0.5	1	≤1000	≤2	0.25	≤0.5	1
M12-85	19	012	08/02/12	1	tNew^b^	8	Pos	>8	>6	8	>2	≤0.5	≤0.5	>4	≤2	>4	2	2	≤32	>4	>8	≤0.5	≤0.5	≤1000	≤2	0.25	≤0.5	1
M12-117	19	011	17/02/12	1	tNew^b^	8	Pos	>8	>6	8	>2	≤0.5	≤0.5	>4	≤2	>4	2	2	≤32	>4	>8	≤0.5	≤0.5	≤1000	≤2	0.25	≤0.5	1
M12-559	28	005	02/05/12	1	t008	8	Pos	>8	>6	16	>2	≤0.5	2	>4	≤2	>4	4	4	≤32	>4	>8	1	≤0.5	≤1000	≤2	0.25	≤0.5	2
M11-81	05	021B	11/10/11	2	t034	398^c^	Neg	>8	>6	16	≤1	>2	1	>4	4	≤0.25	2	≤0.25	≤32	>4	>8	4	2	≤1000	>16	0.25	≤0.5	1
M11-215	06	017	07/12/11	2	t034	398	Neg	>8	>6	8	≤1	≤0.5	≤0.5	≤0.25	≤2	0.5	2	≤0.25	≤32	>4	>8	≤0.5	≤0.5	≤1000	>16	0.25	≤0.5	1
M12-473	25	010	19/04/12	2	t034	398	Neg	>8	>6	8	≤1	≤0.5	≤0.5	≤0.25	≤2	≤0.25	4	≤0.25	≤32	>4	>8	≤0.5	≤0.5	≤1000	>16	0.25	≤0.5	0.5
M12-474	27	004	18/04/12	2	t034	398	Neg	>8	>6	8	≤1	≤0.5	≤0.5	≤0.25	≤2	≤0.25	2	≤0.25	≤32	>4	>8	≤0.5	≤0.5	≤1000	>16	0.25	≤0.5	0.5
M12-475	27	008	17/04/12	2	t034	398	Neg	>8	>6	8	≤1	≤0.5	≤0.5	≤0.25	≤2	≤0.25	2	≤0.25	≤32	>4	>8	≤0.5	≤0.5	≤1000	>16	0.25	≤0.5	0.5
M12-560	30	012	17/05/12	–a	t267	–a	Neg	>8	>6	8	2	≤0.5	≤0.5	0.5	≤2	0.5	2	≤0.25	≤32	>4	>8	≤0.5	≤0.5	≤1000	≤2	0.12	≤0.5	1
M12-561	30	011	17/05/12	4	t267	97	Neg	>8	>6	8	≤1	≤0.5	≤0.5	0.5	≤2	0.5	2	≤0.25	≤32	>4	>8	≤0.5	≤0.5	≤1000	≤2	0.25	≤0.5	1
M12-562	30	012	16/05/12	4	t267	97	Neg	>8	>6	8	≤1	≤0.5	≤0.5	0.5	≤2	0.5	2	≤0.25	≤32	>4	>8	≤0.5	≤0.5	≤1000	≤2	0.25	≤0.5	1
M12-563	30	003	18/05/12	4	t267	97	Neg	>8	>6	8	≤1	≤0.5	≤0.5	0.5	≤2	0.5	2	≤0.25	≤32	>4	>8	≤0.5	≤0.5	≤1000	≤2	0.25	≤0.5	1
M12-564	30	008	16/05/12	4	t267	97	Neg	>8	>6	8	≤1	≤0.5	≤0.5	0.5	≤2	0.5	2	≤0.25	≤32	>4	>8	≤0.5	≤0.5	≤1000	≤2	0.25	≤0.5	1
M12-120	19	008	16/02/12	3	t002	105	Neg	>8	>6	16	>2	>2	≤0.5	>4	≤2	>4	2	>4	≤32	>4	>8	≤0.5	≤0.5	≤1000	≤2	0.25	≤0.5	1
M12-472	24	010	20/04/12	3	t002	105^c^	Neg	>8	>6	8	>2	≤0.5	≤0.5	>4	≤2	>4	2	>4	≤32	>4	>8	≤0.5	≤0.5	≤1000	≤2	0.25	≤0.5	1

a Not available.

b Three repeat insertion from t008.

c One variant position different from sequence type specified.

d Minimum inhibitory concentration (µg/ml). Breakpoints for antimicrobial susceptibility (S =  sensitive, R =  resistant): Ampicillin: S≤0.25/R≥0.5, Cefoxitin: R>4, Chloramphenicol: S≤8/R≥32, Ciprofloxacin: S≤1/R≥4, Clindamycin: S≤0.5/R≥4, Daptomycin: S≤4/R≥16, Erythromycin: S≤0.5/R≥8, Gentamycin: S≤4/R≥16, Levofloxacin: S≤1/R≥4, Linezolid: S≤4/R≥8, Moxifloxacin: S≤0.5/R≥2, Nitrofurantoin: S≤32/R≥128, Oxacillin +2% NaCl: S≤2/R≥4, Penicillin: S≤0.12/R≥0.25, Quinpristen/dalfopristin: S≤1/R≥4, Rifampin: S≤1/R≥2, Streptomycin: Not available, Tetracycline: S≤4/R≥16, Tigecycline: S≤0.5, Trimethoprim/Sulphamethoxazole: S≤2/R≥4, Vancomycin: S≤2/R≥16.

Of the 637 rats tested, 601 (94.3%) were Norway rats and 36 (5.7%) were black rats. None of the black rats were positive for MRSA. Given the low number of black rats trapped and differences in the ecology of brown and black rats, black rats were excluded from further statistical and spatial analyses.

### Statistical analysis

On bivariable analysis, rats caught in the spring and winter had greater odds of being MRSA-positive compared to rats caught in the fall (Table 2). The odds of being MRSA-positive also increased with increasing weight and volume of internal fat. There was no significant association between MRSA-status and length, sex, or sexual maturity. The final model included season and body fat (Table 2).

**Table 2:** Relationship between MRSA-status, season, and morphometric characteristics among a population of wild Norway rats.

Characteristic		MRSA-positive n (%)^a^ n = 22	MRSA-negative n (%)^a^ n = 579	Unadjusted odds ratio (95% CI)	Adjusted odds ratio (95% CI)
**Season**					
	Fall	3 (13.6)	232 (40.1)	Ref^b^	Ref^b^
	Winter	9 (40.9)	126 (40.9)	5.52 (1.62 – 25.52)	5.29 (1.04 – 26.85)
	Spring	10 (45.5)	212 (36.6)	3.65 (1.10 – 16.43)	5.50 (1.10 – 27.58)
	Summer	0 (0)	9 (1.6)	–c	–c
**Sex**					
	Female	11 (50.0)	247 (42.7)	Ref^b^	
	Male	11 (50.0)	324 (56.0)	0.76 (0.32 – 1.81)	
**Maturity**					
	Immature	5 (22.7)	180 (34.1)	Ref^b^	
	Mature	17 (77.3)	348 (65.9)	1.76 (0.68 – 5.42)	
**Weight** (10g)					
	Median (IQR)	20.9 (6.4 – 33.0)	15.7 (6.6 – 26.1)	1.03 (1.00 – 1.07)	
**Length** (cm)					
	Median (IQR)	20.0 (13.1 – 22.0)	18.0 (13.5 – 21.0)	1.07 (0.97 – 1.19)	
**Fat Score**					
	Median (IQR)	2.0 (1.0 – 2.0)	1.0 (0.0 – 2.0)	2.22 (1.27 – 4.16)	2.53 (1.33 – 4.82)

a Frequencies and percentages may not add to 100% because of exclusion of rats with missing data for the variable in question.

b Reference category.

c Insufficient power for accurate estimation.

### MRSA *spa* typing and antibiotic susceptibility

Six different *spa* types were observed, the most common of which was t008 (n = 7), followed by t267 (n = 5), t034 (n = 5), t002 (n = 2), and t818 (n = 1, a four repeat truncation of t008). Two isolates belonged to a *spa* type (‘tNew’) that had never been observed before and represented an insertion of three repeats into t008. Characteristics of the 22 isolates are summarized in Table 1.

### Whole-genome sequencing

**Figure 2:**
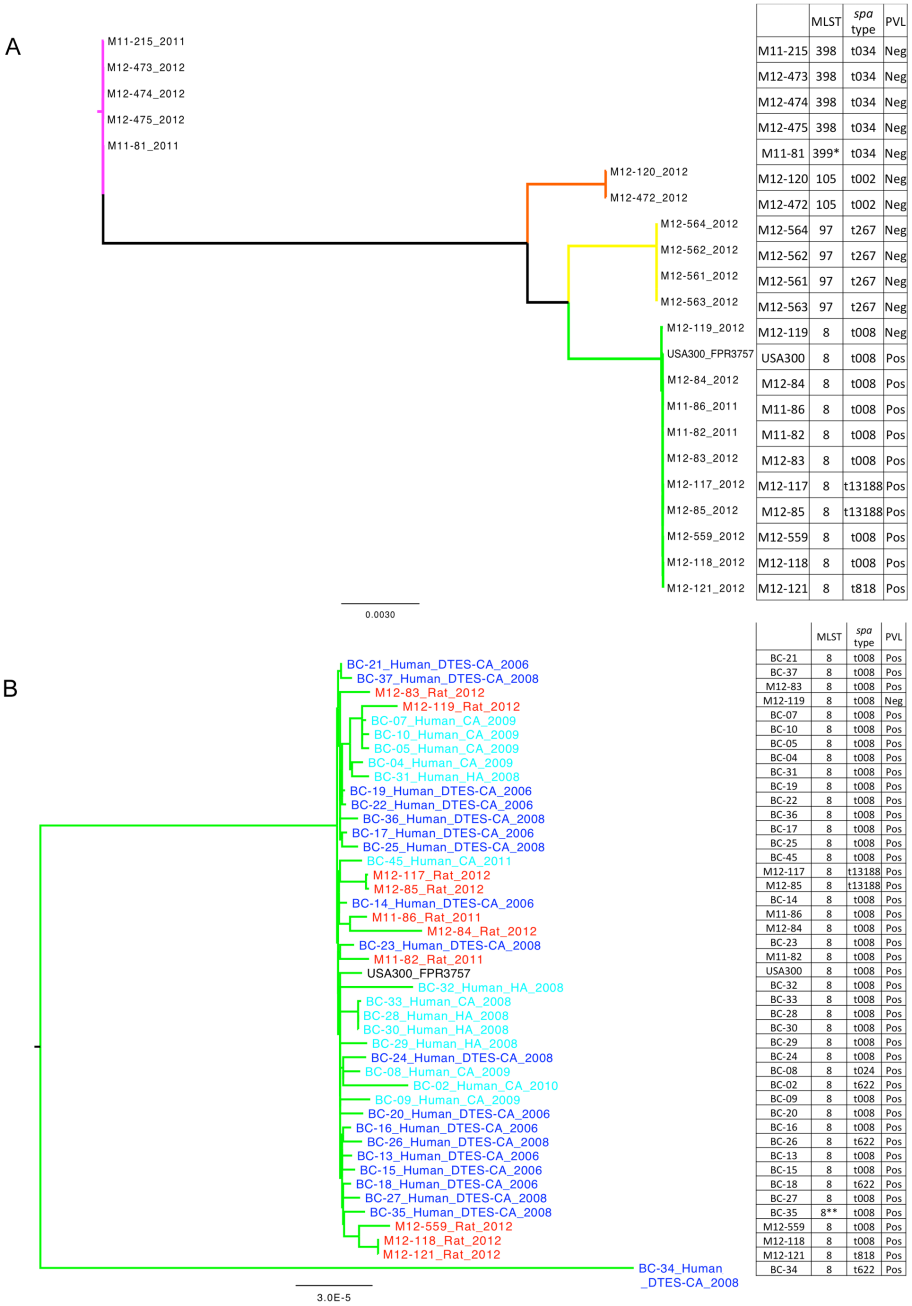
Maximum likelihood phylogenetic tree of all rat MRSA samples. 2a: Branch color represents cluster: Green  =  Cluster 1; Pink  =  Cluster 2; Yellow  =  Cluster 3; Orange  =  Cluster 4. Labels give isolate ID and date. MLST, *spa* type, and presence of PVL shown in right columns. Abbreviations: ST =  Sequence Type; tNew  =  New *spa* type with 3 repeat insertion from t008. Scale bar represents the average number of substitutions per site. 2b: Maximum likelihood phylogenetic tree of all rat and human MRSA ST8 samples. Labels give isolate ID, sample location and date. Text color represents sample isolation location: Red  =  Rat; Dark blue  =  Human DTES; Pale blue  =  Human other. MLST, spa type and presence of PVL shown in right columns. Abbreviations: DTES =  Downtown East Side; CA =  Community-acquired; HA =  Hospital-acquired; ST =  Sequence Type; tNew =  New *spa* type with 3 repeat insertion from t008. Scale bar represents the average number of substitutions per site.

Whole-genome sequencing of the rat MRSA samples showed that they grouped into 4 clusters in accordance with the *spa* typing and multi-locus sequence type (MLST) data (Figure 2a, Figure S1a), the latter of which was generated from the WGS data. Cluster one (n = 10) isolates belonged to sequence type (ST)8 and consisted of three *spa* types: *spa* t008, t818, and ‘tnew’. All but one of these isolates were positive for PVL and consistent with the USA300 clone. Cluster two (n = 5) isolates belonged to ST398/t034, typically referred to as LA-MRSA. Cluster three (n = 4) was ST97/t267. Cluster four (n = 2) consisted of ST105/t002, a clonal complex 5 strain that is classified by PFGE as USA100 or Canadian epidemic MRSA 2 (CMRSA-2). Isolates from clusters 2–4 were PVL negative. There was 100% concordance between in silico and lab based results for PVL and *spa* type.

### Bioinformatics

In general, within 3 of the clusters the isolates were genetically diverse (Figure S2). Isolates in cluster one differed from each other by a mean of 93 variant positions, while those in clusters two and four differed by an average of 53 and 100 variant positions. In contrast, isolates within cluster three had no variant positions between them. However, there were also several similar isolates in clusters one and two. In cluster one, isolates M12-85 and M12-117 had 7 variant positions between them, and isolates M12-121 and M12-118 had 2 variant positions between them. In cluster two, isolates M12-473, M12-474 and M12-475 had 9–10 variant positions between them.

When DTES-origin human samples were compared to the rat samples in cluster one, the genome sequence results could not distinguish between the two (Figure 2b, Figure S1b). The average pairwise distance among the rat samples and DTES human samples (within group comparison (Π_x_)) was 119 and 104 variant positions respectively, and the average pairwise distance between the rat and DTES human samples (between group comparison (Π_xy_)) was 114 variant positions.

Investigation of non-*S. aureus* regions within the accessory genome revealed a 4 kb region of interest present in all ST97 strains/cluster three with 89% similarity to *Staphylococcus pseudintermedius*, and no matches to *S. aureus* strains in the NCBInr database. However, further investigation revealed that this region has been identified in a ST395 *S. aureus* isolate recently sequenced from Italy [25].

### Spatial analysis

**Figure 3:**
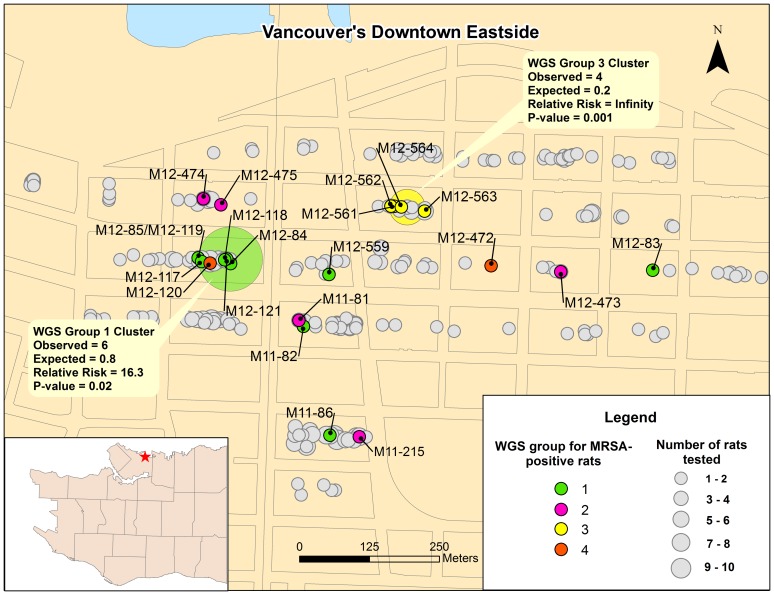
Geographic distribution of MRSA-positive rats. Inset  =  Map of Vancouver with location of study site.

Figure 3 illustrates the distribution of MRSA-positive rats, including WGS cluster for positive rats. Two distinct clusters of higher than expected MRSA prevalence were identified, 1 for cluster one and 1 for cluster 3.

Interestingly, isolates M12-561, M12-562, M12-563, M12-564, which had no variant positions between them, all originated from the same city block (block 30). Isolates M12-85 and M12-117, which had 7 variant positions between them, and isolates M12-121 and M12-118, which had 2 variant positions between them, originated from block 19. Isolates, M12-473 and M12-475, which had 10 variant positions between them, originated from block 27. In contrast, other samples originating from the same block showed relatively high genetic diversity.

## Discussion

This study demonstrates that urban rats carry a variety of MRSA lineages previously described in humans and livestock. More specifically, four distinct genetic clusters were identified in the rat population under study.

Four of the isolates were assigned to cluster three, which corresponds to ST97, an MRSA clone that has been reported in both people and livestock [26]–[28]. Five isolates were assigned to cluster two, which corresponds to the LA-MRSA strain ST398 [8]. Interestingly, both ST97 and ST398 have been previously identified in rats collected from livestock farms [11]. The origin of these strains in our study area, however, is unclear. ST398 is known to colonize and infect humans, but is much more prevalent in production animals [8], suggesting that the most likely source of this ST was contaminated animal products [29]. Groceries, restaurants, and other premises that process, prepare, and sell animal products are common throughout the study area, so exposure through infestation of these premises or through waste scavenging is possible. Although ST97 originated in livestock, it is now recognized to be an emerging pandemic clone of CA-MRSA [28], making it difficult to speculate as to the reservoir of this ST.

Other isolates were more clearly of human origin. For example, 2 isolates were assigned to cluster four, which corresponds to ST105. This ST belongs to clonal complex 5 (CC5), the leading cause of HA-MRSA infection in Canada [30], as well as CA-MRSA colonization in Canada and the United States [30], [31]. MRSA CC5 has also been reported in various domestic animals in Canada [32].

The most common genetic cluster in our study corresponded to the CA-MRSA strain USA300 [21], the most common strain of MRSA circulating in the DTES human population [14]. Rat isolates from this cluster were genetically indistinguishable from DTES human isolates and had the same degree of genetic diversity. These findings are supportive of multiple transmissions of MRSA between humans and rats, although the direction is unclear and common-source exposure cannot be excluded.

Although spatial clustering is a common characteristic of urban rat populations [33], [34] and the microbes that circulate within them [35], clustering was not a strong feature of MRSA in rats with the exception of ST97. Indeed all MRSA isolates from block 30 that were characterized by WGS were genetically identical and identified as ST97. At this point this point it cannot be determined whether geographic clustering of ST97 represents rat-rat MRSA transmission or exposure to a common ‘non-rat’ source of MRSA within the block.

The statistical analysis suggests that MRSA status (positive vs. negative) in rats is associated with both body fat and season. Body fat is likely a good marker of dominance within a rat colony, as dominant rats have the greatest access to food resources [34], [35]. Dominant rats are also more likely to explore new objects and territories and to engage in social interactions (e.g., fighting and mating) [34], which may increase their likelihood of being exposed to MRSA in other rats or the environment. The association with MRSA carriage and season could reflect temporal variation in rat-rat or rat-human interactions [34], [36].

In addition to their ability to carry MRSA, rats might also act as a ‘mixing vessel’, facilitating transfer of genetic elements between *S. aureus* and other bacterial species. Interestingly, all of the ST97 isolates contained a genetic element identical to that found in *Staphylococcus pseudintermedius*, which has also been identified in this rat population [13]. Unfortunately, the rats from block 30, where the ST97 was found, were not among those tested for *S. pseudintermedius*. Given that this element has been identified in *S. aureus* ST97 from another study [25], it is difficult to confirm its true origin. Regardless, it is important to consider that rats actively explore a number of different human environments, and therefore have a unique opportunity to become exposed to/colonized with a variety of human pathogens and to facilitate gene transfer among these organisms.

Although these results show that rats can carry MRSA, the pathways through which MRSA is acquired by rats, the degree to which MRSA is maintained in rat populations, and the potential significance of rats as a reservoir of MRSA for humans, animals, and the environment remain unclear. One limitation of the present study was the focus on MRSA versus *S. aureus* in general. Inclusion of methicillin-sensitive *S. aureus* in future studies may help to elucidate the ecology of *S. aureus*, including MRSA, in rats. Additionally, we chose to sample the rat nares and oropharynx only, as this was the methodology used in a past study of MRSA in rats [11]. However, given that MRSA can be shed in the feces of other animal species [37], it would be prudent to sample the rectum or feces in future studies to investigate if the same is true for rats, particularly since fecal shedding of MRSA in rats could increase their potential to spread the bacterium.

Previously, studies of disease risks associated with rats have largely focused on pathogens for which rats are the primary reservoir (e.g., e.g., *Leptospira* spp.). However, this study shows that rats can also carry pathogens more commonly associated with humans and other animal species. This is particularly significant with regard to MRSA because: a) MRSA infection is a substantial problem in impoverished urban communities; and b) these communities are also most likely to experience rat infestations and rat-to-human disease transmission. Overall, this study signals the need for additional research regarding the ecology and significance of MRSA carriage in urban rats.

## Supporting Information

Figure S1 **Bootstrap support out of 500 replicates for** **Figure 2** **.**
(TIF)

Figure S2 **Number of variant nucleotide positions between every MRSA sample isolated from rats calculated from reference based assembly.**
(TIFF)
