# Supplementary figures and images for: Carriage of Methicillin-Resistant Staphylococcus aureus by Wild Urban Norway Rats (Rattus norvegicus)
Source: PLoS One. 2014 Feb 3;9(2):e87983. doi: 10.1371/journal.pone.0087983 (PMC3912160; doi:10.1371/journal.pone.0087983)

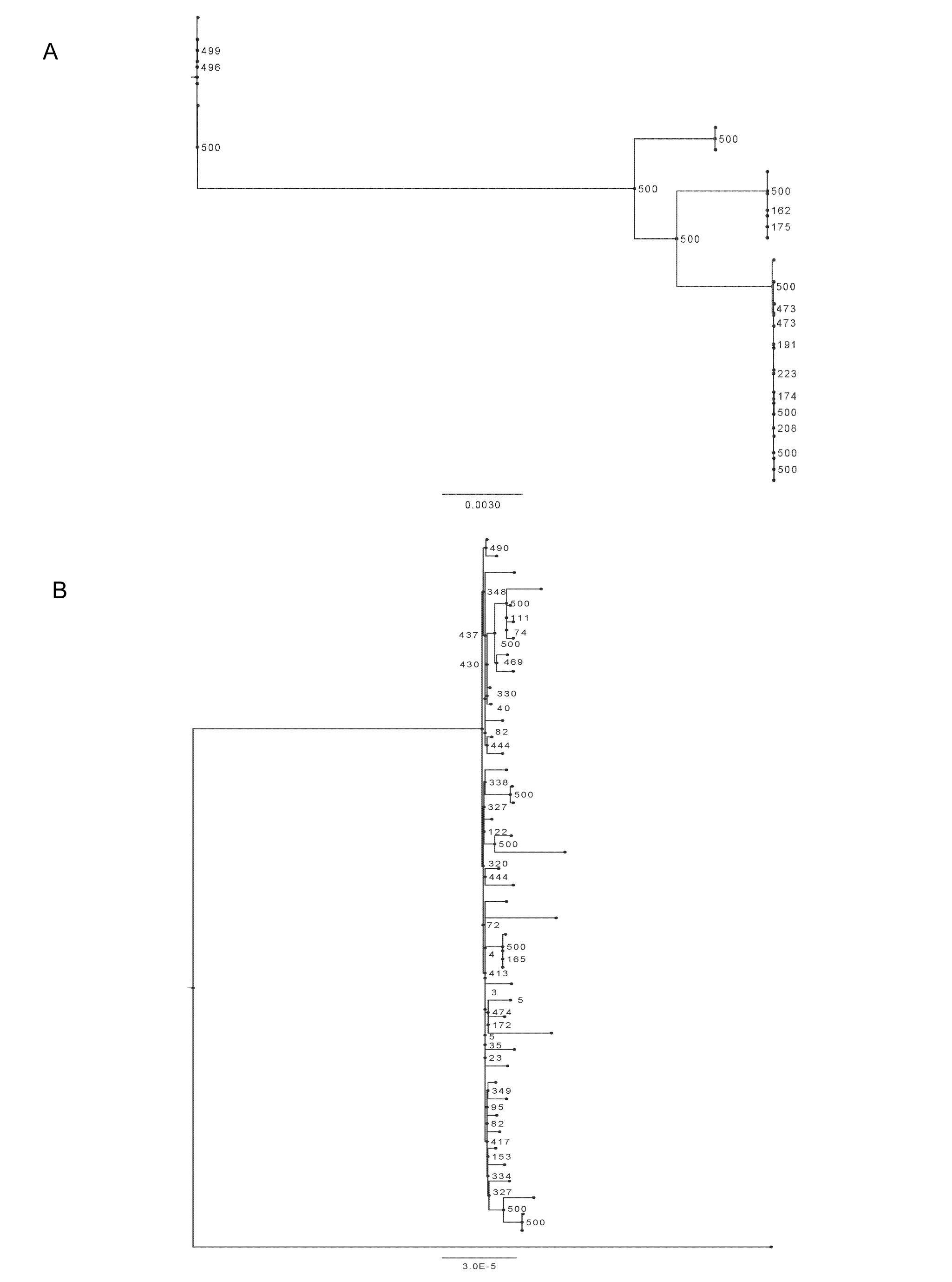

Supplement: Figure S1 — Bootstrap support out of 500 replicates for Figure 2 . (TIF) [file pone.0087983.s001.tif]

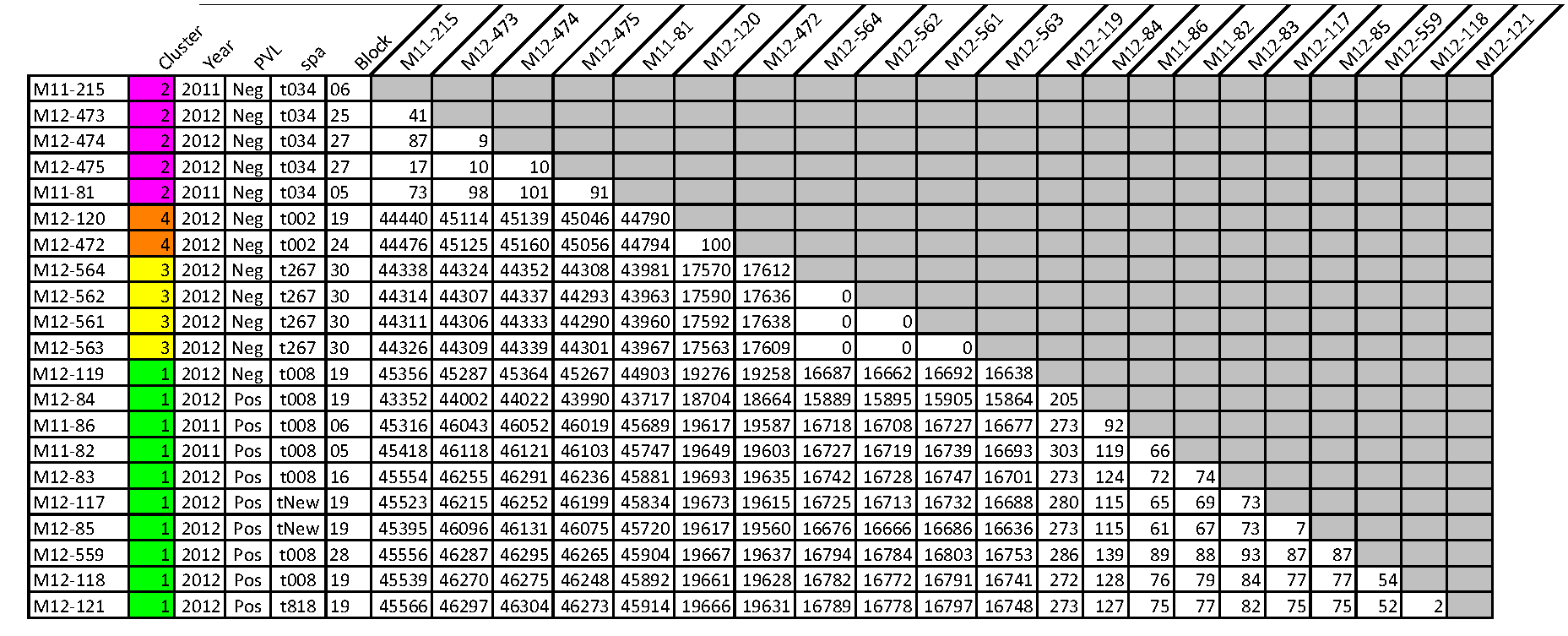

Supplement: Figure S2 — Number of variant nucleotide positions between every MRSA sample isolated from rats calculated from reference based assembly. (TIFF) [file pone.0087983.s002.tif]
